# Exploring differences and similarities of EQ-5D-3L, EQ-5D-5L and WHOQOL-OLD in recipients of aged care services in Germany

**DOI:** 10.1371/journal.pone.0290606

**Published:** 2023-08-25

**Authors:** Ole Marten, Wolfgang Greiner

**Affiliations:** Department of Health Economics and Health Care Management, Bielefeld University, Bielefeld, North Rhine-Westphalia, Germany; University of Bamberg, GERMANY

## Abstract

European countries more than ever face shifts towards aging societies with accompanying challenges for health and aged care services. Economic evaluation has mainly relied on health measures such as EQ-5D across populations and conditions. We want to know how well the EQ-5D performs in the target population to avoid bias to the disadvantage of older adults and care-dependents. Therefore, we aim to explore differences and similarities of EQ-5D-3L and EQ-5D-5L in comparison to the old-age specific WHOQOL-OLD instrument in a sample of older adults receiving aged care services. We collected data from n = 329 older adults (≥65 years) receiving aged care services in Germany; the majority was at least 80 years and had varying care needs. We assessed instruments’ feasibility, test-retest reliability, instruments’ association and sensitivity to known-group differences. In terms of feasibility and test-retest reliability both EQ-5D versions performed better than the WHOQOL-OLD. All measures differentiated well between groups based on aspects of general health and care levels. The analysis of relationship between measures indicated that EQ-5D and WHOQOL-OLD assess partially overlapping, but distinct constructs. We found no clear evidence of superiority of either EQ-5D version over the other. The EQ-5D-5L performed better in terms of test-retest reliability and stronger correlations with WHOQOL-OLD facets. We conclude that using the WHOQOL-OLD alongside EQ-5D in this sample added further information on different aspects of quality of life.

## Introduction

European countries experienced a major increase in life expectancy over the past decades. Recent projections for the countries of the European Union indicate that the share of older people, i.e. those being 65 years and above, is further going to increase from ~21% in 2021 up to 31.3% in 2100. Similarly, the proportion of those aged 80 and over is also expected to increase from 6% to 14.6% during that time [1]. Moreover, the association between aging and diminished health and even multimorbidity is well documented [2, 3] and corresponds to higher health and aged care service utilisation in older adults in comparison to the younger general population [2, 4, 5].

Aged care involves a set of services addressing a person’s health and care needs, which arise as a consequence of reduced functional capacity [6]. These services can be either provided at home, where recipients stay in their familiar living environment, or in institutions such as nursing homes. On the other hand, aged care services may be provided by formal caregivers, who usually have a professional training and provide paid care services, whereas informal care refers to unpaid care provided by people in close contact to the dependent (e.g. partners, children or relatives) [7, 8]. In simple terms, aged care services can be characterised based on where and by whom these services are provided.

In 2021 around 4.96 million people in Germany were dependent on care with 84% being cared for at home. Overall, slightly more than 50% of care dependents exclusively received informal care through relatives and one fifth received a combination of formal and informal care services at home. On the other hand, in 2021 around 16% of care dependents in Germany were living in nursing homes [9]. Due to the great number of older adults and in the perspective of continuously increasing demand for aged care services many complex interventions are being developed to address the needs of older adults. In this population, where interventions may operate at the intersection of health and aged care, the outcomes may well go beyond health. In this regard, the outcome of care may not be restored health or functional ability, but rather improved quality of life (QoL) by helping recipients to participate in activities of daily living or social interaction [10]. While health care and aged care may be related sectors, budgets are strictly separated. Especially with finite resources the accurate measurement of outcomes is of significant importance, since this information is crucial for effective decision-making based on economic evaluation for either sector.

So far, economic evaluation has mainly relied on generic measures of health—such as EQ-5D (hereafter, used to refer to both the EQ-5D-3L and EQ-5D-5L)—as the preferred outcome measure, where this is to ensure comparability of outcome measurement across conditions and all adult age groups. However, if conditions or interventions affect outcomes other than health or if benefits accrue in more than one sector measures of health may be limited in their capacity to fully grasp the important outcomes [11]. The EQ-5D appears to be frequently used in economic evaluation of interventions addressing older adults [12, 13] as well as in aged care settings [8, 14]. In this instance the outcome assessment could be supplemented with an old-age-specific measure of QoL, which can contribute additional profile information. Old-age-specific measures are developed with older people considering what aspects are important to them, where the WHOQOL-OLD is one of the more prominent instruments in this field [15, 16]. With the EQ-5D being a keystone measure for a variety of different populations, but also in older adults and those being dependent on care, we want to know how well the EQ-5D performs in the target population to avoid bias to the disadvantage of older adults and care-dependents. Gottschalk et al. [17] systematically reviewed EQ-5D measurement properties in samples of older adults with an average age ≥75 years and update the work of Haywood and colleagues [18]. Even though the EQ-5D-5L has been around for quite some time [19], the evidence on its measurement properties in older adults is relatively scarce; especially with regard to the assessment of the instrument’s reliability [17, 20]. Furthermore, we are unaware of further studies assessing the relationship of either the EQ-5D-3L or EQ-5D-5L with the old-age specific QoL instrument WHOQOL-OLD in German recipients of aged care services.

The EQ-5D and WHOQOL-OLD are not measuring identical but overlapping constructs, however, if both measures are designed or regularly applied to evaluate interventions for older people and aged care services, then we want to know how well they perform in this context. Therefore, this paper aimed to understand the differences and similarities between EQ-5D-3L, EQ-5D-5L and the old-age specific WHOQOL-OLD instrument in a sample of older adults receiving aged care services by investigating sensitivity to known-group differences and test-retest reliability of the measures as well as by exploring the relationship of the EQ-5D with the WHOQOL-OLD.

## Methods

### Sampling procedure

For this study, we used a convenience sampling approach to recruit respondents. In order to invite participants to the survey, we applied a two-staged contacting procedure. In the first stage, we approached social care providers, e.g. nursing homes, ambulatory care services or adult day-care centres, in the cities of Bielefeld (North Rhine-Westphalia) and Schwerin (Mecklenburg-western Pomerania) as well as their surrounding areas to invite institutions to cooperate in this study. Only in the second stage, potential participants from cooperating institutions were invited to participate in this survey between September 2019 and March 2020. However, respondents were exclusively contacted by their respective care facility to assess their willingness to participate in this study to minimise external and unfamiliar contacts for this potentially vulnerable group.

Participants eligible for this study were i) at least 65 years and ii) in need of aged care services; that involves services that address an individual’s health or personal care needs and that is either given by family members or friends or in a professionalised setting by health and social care workers [21]. Inclusion into the study was based on respondents’ self-reports; no official records were reviewed. The collected data is anonymous and, hence, no formal written consent was required. Ethics approval under the number 2017–207 was obtained from the Research Ethics Committee at Bielefeld University.

### Survey design and instruments

The survey consisted of two different EQ-5D versions, the WHOQOL-OLD as well as a sociodemographic questionnaire. The EQ-5D-3L (3L) is a generic preference-based measure of health-related quality of life comprising two parts. The first is the descriptive system made up of five dimensions: mobility, self-care, usual activities, pain or discomfort and anxiety or depression. Each of the dimensions can be described by one of three severity levels ranging from no problems to extreme problems. The 3L can describe 243 unique health states [22]. The EQ-5D-5L (5L) is a later version of the instrument comprising the same five dimensions. However, in this variant the number of response options was increased to five severity levels representing no, slight, moderate, severe or extreme problems. Thus, the descriptive system differentiates 3125 individual health states [19]. The second component of both the 3L and 5L is the EQ VAS. Respondents are asked to indicate their subjective overall health on this vertical scale, which uses ‘the worst health you can imagine’ and the ‘the best health you can imagine’ as reference points to the scale [23].

The WHOQOL-OLD is an old age-specific measure of quality of life intend for use in adults being 60 years or older. The measure was developed by the WHOQOL group with the aim to generate a measure that is psychometrically sound with older adults and, secondly, covers dimensions that are of importance to older respondents. The WHOQOL-OLD has 24 items that are equally distributed across six facets: sensory abilities; autonomy; past, present and future activities; social participation; death and dying; and intimacy [15]. Each facet can be scored and transformed onto a scale ranging from 0 to 100 allowing the calculation of a profile of scores. In addition, a total score can be computed by averaging across the scores of the six sub-scales. This study uses the German version of the WHOQOL-OLD, which has been shown to be psychometrically valid in the German older population [16].

For the sociodemographic questionnaire, respondents were asked to self-report their gender, age group, educational level, marital status and care setting as well as care level. Care levels were categorised in accordance with the German statutory long-term care insurance [24, 25], where five care levels facilitate the classification of the type and severity of impairments with independence or ability, irrespective of whether these are physical, mental or psychological. To determine the independence of a person needing care, they will be evaluated in the following six modules: “mobility”, “mental and communication-related abilities”, “behaviour and psychological issues”, “self-care”, “independent handling of requirements and challenge associated with illness or therapy–and their management” and “everyday life and social contacts”. Depending on the degree of impairments, they will be assigned to one of five care levels labelled as ‘minor’, ‘considerable’, ‘serious’, severe’ or ‘most severe’ impairments of independence or ability; the assigned care level consequently affects the amount of benefits people in need of care can receive from the statutory long-term care insurance [26]. Further, participants answered a single-item general health status question on a five-point scale (very good, good, moderate, bad, very bad). Lastly, respondents indicated whether they needed help answering the survey and what kind of help they needed.

For each respondent, two surveys were performed, where the second survey was required to assess the test-retest reliability of the WHOQOL-OLD and both EQ-5D versions. Since both the 3L and 5L include an identical visual analogue scale (EQ VAS), we included this component only once. Respondents first answered a version of the EQ-5D including the EQ VAS followed by the WHQOOL-OLD and then continued with the second versions of the EQ-5D. The survey finished with the sociodemographic questionnaire. The presentation order of the EQ-5D versions was varied, so that half of the sample answered the 3L first and then the 5L (after they had finished the WHOQOL-OLD) or vice versa. For the second survey, the sociodemographic questionnaire was dropped to minimise respondent burden.

Cooperating institutions were provided with a bespoke number of survey packages. Each package included an information sheet, the paper-based survey as well as stamped and addressed return envelopes. To assess test-retest reliability of the survey components, the second survey was also included in the survey package, where both the initial and retest survey were marked with a matching four-digit identification number. The retest interval was set to 14 days, assuming that the interval would be short enough so that the health status would be fairly constant, but just long enough to minimise the risk that survey participants recall the questions and their answers [27]. Generally, each of the included instruments was designed as a self-complete measure and applied as such. However, if respondents required help with the survey, they could be assisted.

### Data analysis

The health and QoL information as generated by the 3L, 5L and the WHOQOL-OLD was collected as self-reported data from each participant individually. We derived the interval-scaled summary scores for each respondent and for each instrument using the recommended scoring technique. The European VAS tariff by Greiner et al. [28] was used to calculate the 3L index values, whereas for the 5L variant we applied the tariff by Ludwig et al. [29] to generate the index. To generate the WHOQOL-OLD facet scores and the total score, we used the recommended procedure by Conrad et al. [30].

Feasibility of the included measures was analysed in terms of missing values for the descriptive system for the 3L, 5L and WHOQOL-OLD as well as for the EQ VAS; additionally, we summarise problems that occurred with the EQ VAS. We examine completion rates for all measures based on their summary score. Further, we analysed the amount of time respondents needed to complete the survey as well as the extent and type of aid they required.

Test-retest reliability of interval-scale data, i.e. 3L and 5L indices, EQ VAS score, WHOQOL-OLD facets’ and total scores, were determined using intraclass correlation coefficients (ICCs). In accordance with Koo and Li [31], we used two-way mixed effects models specifying the absolute agreement option. Recommended threshold values for ICCs to categorise reliability are <0.5, 0.5–0.75, 0.75–0.9 and >0.9 representing poor, moderate, good or excellent reliability [31]. The intra-rater reliability of categorical variables, i.e. the EQ-5D descriptive system and individual WHOQOL-OLD items, was assessed using Cohen’s weighted kappa. Landis and Koch [32] suggest the following cut-off points for the kappa values: agreement is classified as slight for values below 0.2, 0.21–0.4 as fair, 0.41–0.6 moderate, 0.61–0.8 substantial and as almost perfect for values above 0.8. Respondents were eligible for the retest analysis, if they indicated no change in their health status, i.e. respondents answered the general health status questions in the initial assessment and at follow-up with the same category [27].

Evidence suggests that health significantly contributes to older adults’ conceptualisation of QoL [33, 34]. In the absence of a ‘gold standard’ for the assessment of QoL in older adults, we evaluated the relationship between EQ-5D and WHOQOL-OLD by correlating EQ-5D dimensions, indices and EQ VAS with the WHOQOL-OLD total score and facet scores. Relationship of the constructs was expressed either as a Pearson correlation coefficient (interval data) or as a Spearman correlation coefficient (ordinal data). According to the guidelines proposed by Cohen [35] correlations below 0.3 were considered as poor, between 0.3 and below 0.5 as moderate and above 0.5 as strong. Since generic health status as assessed by EQ-5D and old-age specific QoL as measured by WHOQOL-OLD are related constructs, we assumed that EQ-5D indices and the WHOQOL-OLD total score demonstrate a positive, moderate and significant correlation. We expected moderate and significant correlations between the EQ-5D physical dimensions mobility, self-care and usual activities and the WHOQOL-OLD facets autonomy, social participation and past, present and future activities. Similarly, we expected a moderate and significant correlation between anxiety or depression and death and dying. However, for the other EQ-5D dimensions we expected poor correlations between death and dying, intimacy or sensory abilities.

Further, we examined sensitivity to known-group differences in EQ-5D index scores (3L and 5L), EQ VAS, WHOQOL-OLD facet scores and total score by subgroups based on gender, educational level, care level and general health status using analysis of variance (ANOVA) and t-test. For the two group comparison we report Cohen’s d as an effect size measure, whereas for more than two groups we report eta squared. Cohen’s d was interpreted to the thresholds: small (0.2–0.49), medium (0.5–0.79) and large (>0.79). Whereas thresholds for eta squared after ANOVA were categorised as small (0.01–0.059), medium (0.06–0.139) and large (>0.139) [35]. We hypothesised that being male, lower education, a higher care level and a lower general health status would result in lower health or QoL scores. For all analyses we use STATA 17 [36].

## Results

### Sample characteristics

Questionnaires were sent to 800 persons with the help of 43 cooperation institutions. In summary, 334 persons returned the survey. Of these, five respondents were younger than 65 years and, hence, were excluded.

Table 1 provides an overview over the sample characteristics. The initial questionnaire was available from 329 eligible respondents, whereas information from the retest survey was provided from 266 respondents (81%). However, the eligible retest analysis sample consists of 168 respondents who indicated no change on the health transition question. The sample was predominantly female and more than 65% were older than 80 years. No respondent was above 100 years. One fifth of the respondents indicated to have no officially assigned care level. Moreover, respondents with high intensity care needs—as represented by care levels 4 and 5—only accounted for 10.9% of the sample. About 60% of the sample received long-term care and support in a home-based setting, i.e. respondents were living in their homes receiving care from their family/relatives, an ambulatory care service or visited an adult day-care centre. The remaining respondents were either living in a nursing home or did not provide this information. Further, the information on the care setting was recoded to represent subgroups that received only formal or informal care. In total, 80 survey respondents were living at home receiving care only from unpaid family members or relatives, whereas 123 respondents were living in a nursing home receiving care from professional health and social care workers. The distinction in the care setting between ‘where’ and by ‘whom’ people receive care was necessary, as we observed a high degree of mixed care arrangements including both formal and informal care components.

**Table 1:** Summary of sample characteristics.

Characteristics		Sample (test)		Retest sample	
		N	%	N	%
**Gender**	Female	215	65.4%	105	62.5%
	Male	98	29.8%	57	33.9%
	missing	16	4.9%	6	3.6%
**Age group**	65–70 years	27	8.2%	16	9.5%
	71–75 years	21	6.4%	13	7.7%
	76–80 years	62	18.8%	35	20.8%
	81–85 years	78	23.7%	41	24.4%
	86–90 years	88	26.8%	40	23.8%
	91–95 years	40	12.2%	18	10.7%
	96–100 years	9	2.7%	4	2.4%
	missing	4	1.2%	1	0.6%
**Marital status**	Single	22	6.7%	11	6.6%
	Married/partnership	100	30.4%	58	34.5%
	Widowed	176	53.5%	85	50.6%
	Divorced/separated	24	7.2%	10	6.0%
	missing	7	2.3%	4	2.4%
**Educational level**	Low	183	55.6%	96	57.1%
	Middle	77	23.4%	40	23.8%
	High	62	18.8%	30	17.9%
	missing	7	2.1%	2	1.2%
**Care level**	0	61	18.5%	34	20.2%
	1	22	6.7%	15	8.9%
	2	99	30.1%	51	30.4%
	3	90	27.4%	45	26.8%
	4	29	8.8%	14	8.3%
	5	7	2.1%	3	1.8%
	missing	21	6.4%	6	3.6%
**Care setting**	Home based	196	61.4%	111	67.3%
	Nursing home	123	38.6%	54	32.7%
	Formal care	123	60.6%	54	51.9%
	Informal care	80	39.4%	50	48.1%

### Feasibility

As Table 2 indicates, the observed share of missing values for all EQ-5D dimensions was well below 4%. The proportion of missing responses on the WHOQOL-OLD items were mostly below 5%; except for item 12 (‘Satisfied with opportunities to continue archiving’), which was missing in 5.8% of respondents. Completion rates for all instruments were fairly high; the index score could be calculated in 94.2%, 96.4% and 97.2% of the cases for the 5L, 3L and the WHOQOL-OLD total score, respectively. Problems with answering the EQ VAS were prevalent for 31% of respondents. In 25.5% of all EQ VAS responses the required mark on the scale was not placed, while just 2.7% of the responses were missing. Other problems such as reporting a range, mismatching values in the box and on the scale account for the remaining 3%.

**Table 2:** Proportion of missing values per dimension/item for the EQ-5D-5L, EQ-5D-3L and WHOQOL-OLD.

**EQ-5D-5L**			**Missing**	**EQ-5D-3L**			**Missing**
**Mobility**			2.1%	Mobility			2.1%
**Self-care**			3.0%	Self-care			1.8%
**Usual activities**			3.7%	Usual activities			2.1%
**Pain/ Discomfort**			2.7%	Pain/ Discomfort			1.2%
**Anxiety/ Depression**			3.3%	Anxiety/ Depression			1.5%
**WHOQOL-OLD facet**	Item number	Item text	**Missing**	**WHOQOL-OLD facet**	Item number	Item text	**Missing**
**Sensory Abilities**	1	Impairments to senses affect daily life	1.8%	**Social Participation**	14	Have enough to do each day	3.0%
	2	Loss of sensory abilities affect participation in activities	2.4%		16	Satisfied with the way you use your time	3.3%
	10	Problems with sensory functioning affect ability to interact	1.5%		17	Satisfied with level of activity	3.0%
	20	Rate sensory functioning	2.1%		18	Satisfied with opportunity to participate in community	2.4%
**Autonomy**	3	Freedom to make own decisions	2.1%	**Death and dying**	6	Concerned about the way you will die	3.7%
	4	Feel in control of your future	3.7%		7	Afraid of not being able to control death	3.3%
	5	People around you are respectful of your freedom	4.3%		8	Scared of dying	3.3%
	11	Able to do things you’d like to	2.1%		9	Fear pain before death	3.3%
**Past, present and future activities**	12	Satisfied with opportunities to continue archiving	5.8%	**Intimacy**	21	Feel a sense of companionship in life	3.3%
	13	Received the recognition you deserve in life	3.7%		22	Experience love in life	3.7%
	15	Satisfied with what you’ve achieved in life	2.1%		23	Opportunities to love	2.4%
	19	Happy with things to look forward to	3.0%		24	Opportunities to be loved	4.0%

In total, 298 respondents reported a valid completion time for the survey; this was calculated as the difference between reported start and end times. The average time to complete the survey was 28.4 minutes with a range of 5 to 165 minutes. The mean completion time of those completing the survey without help did not differ from those who required help. We were not able to calculate separate times for the completion of individual parts, e.g. for the 5L, as only the total time was available.

Over half of the respondents (59%; n = 194) indicated that they required help completing the survey. Of those, 81% needed help from someone reading the questions to them and 79.4% had the responses filled in for them. Cross-tabulation of these two aid types showed that 77.2% (n = 139 out of 180) needed assistance with both. Further, 8.8% reported to need help with translations. However, it remains unclear, if translations were required into a foreign language or, if this was meant in terms of age-adequate language.

### Test-retest reliability

Of those who a returned retest survey, only 64.9% (n = 168) were eligible for the retest analysis, i.e. those indicating no change in health status as assessed by the general health status question. The average time interval between both measurements was 17 days (median 14 days) with a minimum of 7 days and a maximum of 75 days for the retest sample. There was no significant difference in the mean interval between test and retest between respondents who were eligible for the retest analysis and those who were not included in the analysis.

Agreement as measured by Cohen’s weighted kappa was substantial for all 3L dimensions except for usual activities, which can be rated moderate (see Table 3). In case of the 5L, reliability was rated as substantial for all dimensions. Overall, reliability of the 5L was similar or better than that of the 3L with the exception of mobility, where the weighted kappa was slightly lower. The agreement of the interval-scaled EQ-5D indices can be categorised as good for the 3L (0.86) and excellent in the case of the 5L (0.91) as assessed by ICCs. Reliability of responses was lower for the EQ VAS, but still moderate.

**Table 3:** Test-retest reliability of the EQ-5D-3L and EQ-5D-5L.

Dimension	EQ-5D-3L	EQ-5D-5L
	Weighted Kappa	Weighted Kappa
**Mobility**	0.76	0.73
**Self-care**	0.75	0.75
**Usual activities**	0.59	0.65
**Pain/discomfort**	0.61	0.65
**Anxiety/depression**	0.62	0.63
**Intraclass correlation coefficient**		
**EQ-5D index**	0.86	0.91
**EQ VAS**	0.70	

Weighted kappa values for WHOQOL-OLD items were generally lower than those of both EQ-5D versions. These ranged from 0.39–0.65 suggesting fair to substantial agreement. It appears that agreement of items in the autonomy and past, present and future activities facets are consistently lower than others. ICCs of facet scores resemble this pattern; while agreement on these two facets is moderate, it is considered good on the other four facets. Similarly, the ICC on the WHOQOL-OLD total score is 0.83, thus also being considered good (see Table 4).

**Table 4:** Test-retest reliability of WHOQOL-OLD items, facets and total score.

WHOQOL-OLD facet	Item number	Item text	Weighted kappa	WHOQOL-OLD facet	Item number	Item text	Weighted kappa
**Sensory Abilities**	1	Impairments to senses affect daily life	0.58	**Social Participation**	14	Have enough to do each day	0.62
	2	Loss of sensory abilities affect participation in activities	0.59		16	Satisfied with the way you use your time	0.57
	10	Problems with sensory functioning affect ability to interact	0.56		17	Satisfied with level of activity	0.51
	20	Rate sensory functioning	0.62		18	Satisfied with opportunity to participate in community	0.50
**ICC**	**0.79**			**ICC**	**0.77**		
**Autonomy**	3	Freedom to make own decisions	0.48	**Death and dying**	6	Concerned about the way you will die	0.64
	4	Feel in control of your future	0.41		7	Afraid of not being able to control death	0.57
	5	People around you are respectful of your freedom	0.39		8	Scared of dying	0.63
	11	Able to do things you’d like to	0.43		9	Fear pain before death	0.65
**ICC**	**0.57**			**ICC**	**0.78**		
**Past, present and future activities**	12	Satisfied with opportunities to continue archiving	0.46	**Intimacy**	21	Feel a sense of companionship in life	0.52
	13	Received the recognition you deserve in life	0.44		22	Experience love in life	0.62
	15	Satisfied with what you’ve achieved in life	0.49		23	Opportunities to love	0.54
	19	Happy with things to look forward to	0.39		24	Opportunities to be loved	0.57
**ICC**	**0.59**			**ICC**	**0.79**		
**ICC Total score 0.83**							

### Relationship between EQ-5D and WHOQOL-OLD

The Spearman correlation coefficients between comparable dimensions and facets (EQ-5D Mobility, Self-care, Usual activities and WHOQOL-OLD facets autonomy, past, present and future activities and social participation) showed significant and mostly moderate correlations. The pain or discomfort and anxiety or depression dimensions showed significant but weak correlation with the WHOQOL-OLD facets. Similarly, the facets intimacy (not significant), death and dying and sensory abilities had negligible or weak correlation with almost any of the EQ-5D dimensions (Table 5). Comparably and at the summary score level, Pearson’s correlation coefficients between EQ-5D indices or EQ VAS and the WHOQOL-OLD total score and facet scores followed that pattern consistently; facets that were dissimilar to the EQ-5D contents such as intimacy, death and dying and sensory abilities had negligible or weak correlations with the EQ-5D indices or EQ VAS. In turn, the WHOQOL-OLD total score as well as the facets autonomy, past, present and future activities and social participation showed significant and moderate correlations with the EQ-5D indices and EQ VAS (Table 5).

**Table 5:** Relationship of EQ-5D and WHOQOL-OLD; Spearman correlation coefficients of EQ-5D dimensions and Pearson correlation coefficients of EQ-5D indices and EQ VAS with WHOQOL-OLD summary and facet scores.

Dimension	Version	WHOQOL-OLD total score	Sensory abilities	Autonomy	Past, present and future activities	Social participation	Death and dying	Intimacy
**Mobility**	3L	-0.270**	-0.143	-0.215**	-0.215**	-0.314**	-0.102	-0.052
	5L	-0.346**	-0.286**	-0.297**	-0.276**	-0.397**	-0.070	-0.061
**Self-care**	3L	-0.293**	-0.195**	-0.323**	-0.204**	-0.309**	-0.052	-0.086
	5L	-0.350**	-0.218**	-0.346**	-0.258**	-0.394**	-0.065	-0.135*
**Usual Activities**	3L	-0.376**	-0.255**	-0.326**	-0.326**	-0.420**	-0.071	-0.089
	5L	-0.442**	-0.320**	-0.446**	-0.348**	-0.513**	-0.112	-0.024
**Pain/discomfort**	3L	-0.229**	-0.154**	-0.132*	-0.159**	-0.202**	-0.138*	-0.112
	5L	-0.236**	-0.205**	-0.153**	-0.158**	-0.269**	-0.162**	0.015
**Anxiety/depression**	3L	-0.345**	-0.242**	-0.204**	-0.250**	-0.263**	-0.284**	-0.099
	5L	-0.406**	-0.283**	-0.275**	-0.274**	-0.275**	-0.353**	-0.117*
**EQ-5D index**	3L	0.425**	0.250**	0.348**	0.331**	0.418**	0.174**	0.122*
	5L	0.396**	0.257**	0.331**	0.300**	0.435**	0.166**	0.041
**EQ VAS**	n/a	0.400**	0.303**	0.268**	0.308**	0.411**	0.177**	0.053

** p < 0.01

* p < 0.05

### Sensitivity to known group differences

Sensitivity to known-group differences based on EQ-5D and WHOQOL-OLD components with regard to subgroups based on gender, education, care levels and general health status are displayed in Table 6. For the remaining subgroup characteristics Table 6 only provides a descriptive summary based on mean summary scores. The EQ-5D indices and EQ VAS significantly and to a similar extent discriminate between different care levels and categories of general health status with medium to large effect sizes, where higher care levels, i.e. more extensive care needs, and lower general health resulted in lower scores. Similarly, the WHOQOL-OLD total score and facet scores were able to detect significant differences between these groups with mostly medium effect sizes. Exceptions were found in the intimacy and death and dying facets, where group differences were not significant. Differences between groups based on gender and education were mostly not significant; however, few exceptions were observed for subgroups based on education for WHOQOL-OLD total score and autonomy, past, present and future activities and intimacy facets with small effect sizes.

**Table 6:** Mean values and standard deviation for EQ-5D-3L, EQ-5D-5L, EQ VAS and WHOQOL-OLD total as well as facet scores for the total sample and by subgroups.

Characteristics		N	5L index		3L index		EQ VAS		WHOQOL-OLD total score		Sensory abilities		Autonomy		Past, present and future activities		Social participation		Death and dying		Intimacy	
			Mean	SD	Mean	SD	Mean	SD	Mean	SD	Mean	SD	Mean	SD	Mean	SD	Mean	SD	Mean	SD	Mean	SD
**Total sample**		329	0.64	0.30	0.54	0.24	57.2	19.8	62.7	13.3	62.7	22.0	59.4	21.0	63.8	16.6	57.3	19.9	64.3	27.5	68.1	21.2
**Gender**			d		d		d		d		c		d		d		d		d		d	
	Female	215	0.64	0.29	0.53	0.24	57.8	19.5	63.8	13.3	64.8	23.0	60.8	21.5	64.9	15.7	58.4	20.1	65.1	27.6	68.7	20.3
	Male	98	0.65	0.31	0.56	0.24	55.3	20.8	61.6	12.5	59.8	19.7	57.9	20.0	63.0	17.5	54.7	19.5	64.8	25.9	68.3	22.5
	*Cohen’s d*		*0*.*04*		*0*.*17*		*0*.*13*		*0*.*17*		*0*.*23*		*0*.*14*		*0*.*12*		*0*.*18*		*0*.*01*		*0*.*02*	
**Age group**			d		d		d		d		d		d		c		d		b		d	
	65–70 years	27	0.58	0.35	0.47	0.31	50.6	25.4	61.5	14.8	68.0	29.1	64.6	23.0	56.6	14.9	54.7	20.1	55.5	33.9	68.9	20.0
	71–75 years	21	0.61	0.32	0.57	0.26	61.6	18.9	61.6	17.0	63.0	23.4	55.7	25.3	60.6	21.8	51.7	26.3	69.0	25.6	71.7	22.2
	76–80 years	62	0.69	0.28	0.58	0.24	61.7	20.2	62.3	12.6	65.3	19.1	56.8	20.1	64.7	16.7	57.4	19.6	61.1	27.4	67.4	23.2
	81–85 years	78	0.66	0.28	0.55	0.25	55.4	19.3	61.5	11.4	61.5	19.2	60.1	17.5	63.8	14.8	55.1	17.8	59.4	26.4	68.4	18.4
	86–90 years	88	0.62	0.34	0.53	0.24	58.5	18.5	64.9	13.2	62.3	21.5	61.1	22.1	66.6	17.1	60.9	20.7	70.3	23.9	67.6	21.9
	91–95 years	40	0.66	0.24	0.55	0.20	53.5	19.5	64.4	13.1	61.7	25.0	60.4	20.9	66.0	15.1	58.7	18.6	71.2	29.8	68.4	20.3
	96–100 years	9	0.55	0.22	0.43	0.20	52.8	18.6	53.4	17.6	45.8	27.4	47.2	25.4	53.5	17.4	54.2	19.3	54.2	33.6	65.3	27.3
**Marital status**			c		b		d		d		d		b		d		d		d		a	
	Single	22	0.78	0.16	0.66	0.16	56.3	16.0	59.3	14.1	69.0	21.9	61.6	21.2	59.2	19.8	51.5	17.5	67.3	26.5	46.4	24.4
	Married/partnership	100	0.64	0.30	0.54	0.27	55.5	20.6	62.1	12.2	60.3	21.6	55.6	19.6	63.0	16.7	55.2	19.3	60.7	27.8	77.2	19.0
	Widowed	176	0.62	0.31	0.52	0.24	57.9	20.3	63.3	14.0	62.6	22.9	59.9	22.0	65.4	16.3	59.1	20.4	65.7	27.5	66.5	19.0
	Divorced/separated	24	0.73	0.23	0.62	0.21	61.1	16.4	66.0	10.0	68.5	18.3	71.1	16.5	62.5	15.0	60.9	18.3	72.0	23.4	62.2	22.0
**Educational level**			d		d		d		b		d		b		b		d		d		a	
	Low	183	0.62	0.31	0.52	0.24	56.1	19.9	61.0	13.2	62.4	20.7	56.3	22.1	61.5	17.0	56.2	20.6	64.9	27.9	64.1	21.4
	Middle	77	0.70	0.24	0.58	0.24	59.6	19.7	64.4	13.3	64.5	24.1	63.1	20.9	65.7	15.4	58.9	19.3	64.4	25.0	70.1	20.8
	High	62	0.66	0.28	0.56	0.24	57.5	19.8	65.8	13.0	62.9	23.1	64.1	16.9	68.9	15.6	58.9	18.4	62.5	28.9	76.6	18.2
	*Eta squared*		*0*.*012*		*0*.*01*		*0*.*005*		*0*.*024*		*0*.*001*		*0*.*029*		*0*.*032*		*0*.*005*		*0*.*001*		*0*.*053*	
**Care level**			a		a		a		a		b		a		b		a		d		c	
	0	61	0.83	0.14	0.71	0.17	66.9	17.7	68.1	11.9	65.3	18.7	68.3	17.3	69.7	15.0	68.4	16.2	62.4	25.4	74.6	20.3
	1	22	0.75	0.24	0.67	0.18	61.5	18.3	64.1	10.5	70.5	21.2	65.0	17.9	65.2	10.3	58.8	19.6	59.8	24.8	66.2	18.0
	2	99	0.60	0.26	0.50	0.21	55.9	19.0	60.2	14.0	59.5	22.4	55.1	22.3	62.6	17.4	55.5	19.2	63.2	28.8	65.0	23.2
	3	90	0.59	0.29	0.49	0.23	52.4	19.1	61.1	12.7	62.7	22.7	58.4	18.8	61.1	17.8	52.0	19.6	65.2	27.1	66.7	21.5
	4	29	0.50	0.34	0.39	0.27	53.8	19.4	61.6	12.0	60.3	20.5	54.1	21.4	63.2	13.6	54.5	20.8	65.4	18.6	71.2	18.1
	5	7	0.28	0.61	0.32	0.33	32.9	26.7	49.3	14.4	41.1	23.6	41.4	21.6	55.4	22.9	34.8	24.7	62.2	34.1	60.7	23.6
	*Eta squared*		*0*.*16*		*0*.*20*		*0*.*11*		*0*.*075*		*0*.*041*		*0*.*08*		*0*.*042*		*0*.*12*		*0*.*003*		*0*.*032*	
**Care setting**			d		d		d		b		b		c		c		c		a		b	
	Home based	196	0.62	0.32	0.53	0.25	55.7	20.7	61.0	13.1	59.9	21.4	57.6	20.8	62.1	16.8	55.1	20.0	60.1	26.9	70.4	20.6
	Nursing home	123	0.65	0.26	0.55	0.23	58.6	18.5	64.8	12.9	66.6	22.6	61.7	21.5	65.7	15.9	59.3	19.4	71.6	26.5	64.2	21.8
			d		d		d		d		d		d		c		d		b		b	
	Formal care	123	0.65	0.26	0.55	0.23	58.6	18.5	64.8	12.9	66.6	22.6	61.7	21.5	65.7	15.9	59.3	19.4	71.6	26.5	64.2	21.8
	Informal care	80	0.68	0.29	0.58	0.25	59.2	20.0	62.3	11.6	61.9	20.5	57.0	19.8	61.7	14.8	56.1	18.7	63.1	27.1	73.2	21.0
**General health status**			a		a		a		a		a		a		a		a		a		d	
	Very good	9	0.82	0.22	0.71	0.28	73.1	17.1	67.1	13.9	64.1	28.9	56.5	30.6	64.1	16.3	67.2	20.8	75.0	19.2	75.0	16.8
	Good	97	0.82	0.16	0.69	0.20	70.9	16.2	69.4	10.4	70.5	21.5	67.5	19.5	69.1	14.7	64.4	19.2	73.7	20.8	70.1	20.3
	Moderate	174	0.64	0.24	0.53	0.19	55.0	15.4	62.3	12.5	62.1	20.4	59.6	19.4	64.4	15.5	57.9	17.4	61.8	28.5	67.9	20.6
	Bad	47	0.29	0.33	0.26	0.19	35.4	18.0	50.4	11.8	51.1	21.1	43.9	19.5	50.7	18.3	39.7	19.1	55.4	30.0	63.1	25.1
	Very bad	1	-	-	-	-	-	-	-	-	-	-	-	-	-	-	-	-	-	-	-	-
	*Eta squared*		*0*.*37*		*0*.*34*		*0*.*36*		*0*.*21*		*0*.*098*		*0*.*13*		*0*.*12*		*0*.*17*		*0*.*07*		*0*.*016*	

^a^ p < 0.001

^b^ p < 0.05

^c^ p < 0.1

^d^ not significant

With regard to the different care settings the results are just of descriptive nature, since causality of the effect of the care setting on health status or QoL cannot be inferred; here, the EQ-5D indices and EQ VAS did not show significant difference between these subgroups. In turn, significant differences between home based and institutionalised care-recipients were found on the WHOQOL-OLD total score as well as on the sensory abilities, death and dying and intimacy facets. Then again, when looking at the differences between formal and informal care recipients, significant differences were only observable on two facets, where recipients of formal care express less fear of death and dying, and informal care recipients report a higher level of intimacy.

## Discussion

To the best of our knowledge, this is the first prospective study to assess similarities and differences between the EQ-5D versions and the WHOQOL-OLD in a sample of aged care service recipients. The aim of this study was to assess and compare the relationship of instruments, their sensitivity to known-group differences, feasibility and test-retest reliability across EQ-5D and WHOQOL-OLD. In terms of feasibility and test-retest reliability both EQ-5D versions performed better than the WHOQOL-OLD. All three measures were sensitive to known-group differences based on aspects of general health status and care level. The analysis of association between measures indicated that EQ-5D and WHOQOL-OLD assess partially overlapping, but distinct constructs. When comparing the properties of the 3L and 5L in this sample, we found no superiority of either measure over the other. The 5L seemed to do better in terms of test-retest reliability and had stronger correlations with WHOQOL-OLD facets.

The calculated mean summary scores for the different types of care indicate higher QoL on almost all WHOQOL-OLD facets for recipients of formal care or respondents living in nursing homes. An exception to this rule is the intimacy facet, which suggests that respondents receiving informal care report a higher sense of companionship. With regard to the EQ index and EQ VAS results were less clear. Again, mean EQ-5D index and EQ VAS values were higher for the residential care subgroup, which was in line with the reported values on the WHOQOL-OLD facets, however, the opposite was found for the informal care subgroup, who reported slightly higher mean values. These results were contradictory to an earlier study. Borowiak and Kostka [37] used the 3L to compare QoL of older adults living in the community and in aged care institutions and found consistently higher values and fewer reported problems in the community-dwelling subgroup. Hence, we assume that our two-staged sampling process resulted in an underrepresentation of severely ill and highly dependent respondents living in care homes. However, the difference in mean general health status between respondents living at home or in institutions was small. Nevertheless, the direction of causality between health and care provision remains unclear. It could be either way that an individual’s health or QoL status determines the type of provided care or that the type of care has an impact on the individual’s health or QoL status.

With regard to sensitivity to known-group differences we found that both EQ-5D indices and EQ VAS discriminate well between different needs of care, i.e. care levels, which is in line with findings from Hara and colleagues [38] for the Japanese version of the 5L. Similarly, we observed satisfactory ability to differentiate different levels of general health status, which was also confirmed based on the 3L index for older adults in the literature [39, 40]. The WHOQOL-OLD total score and facets performed equally well differentiating subgroups based on their care levels and general health status. We only identified one study assessing and confirming known-groups validity of the WHOQOL-OLD, which assessed differences in general health [41]. These findings confirm our hypotheses with regard to care levels and general health status. On the other hand, we were not able to confirm our hypotheses with regard to group differences based on gender and education.

In the context of aged care services, the imperfect assessment of care needs and care provision makes it difficult to formally state hypotheses and analyse known group differences. Hence, we only reported mean group values based on EQ-5D and WHOQOL-OLD summary scores on a descriptive basis. For EQ-5D indices and EQ VAS we were not able to differentiate subgroups based on different care settings, which was also observed elsewhere [39]. Then again, non-health facets of the WHOQOL-OLD indicated differences in mean values between different care settings at a significant level, which is in line with the broader QoL scope of the measure and the specific target population of older adults.

Moreover, assessing the relationship between the EQ-5D versions and the WHOQOL-OLD, we only observed poor to moderate correlations. At both the individual dimension level as well as for indices or summary scores our hypotheses for central health-related aspects of the EQ-5D with WHOQOL-OLD facets, viz. physical and social functioning (EQ-5D Mobility, Self-care, Usual activities and WHOQOL-OLD facets autonomy, past, present and future activities and social participation), were largely confirmed. However, we had to reject our hypotheses on the correlation with mobility and self-care with the past, present and future activities facet as well for the correlation of mobility with the autonomy facet. On the other hand, our hypotheses with regard to non-health facets of the WHOQOL-OLD were confirmed; as expected, correlations of the facets death and dying and intimacy with all EQ-5D components except anxiety or depression were found to be poor. This suggests that EQ-5D and WHOQOL-OLD capture distinct aspects as indicated by the low correlation. We may conclude that the WHOQOL-OLD assess additional information, which would remain undetected by the EQ-5D. Hence, the best practice model of using additional age-specific measures alongside EQ-5D may be advisable in this population.

This study contributes to the scarce information on the feasibility properties of the 5L in samples of older adults as evidence suggests [20]. Generally, the proportion of missing values for both 3L and 5L was below 4%, which is very consistent with references found for the EQ-5D in the general population as well as in older adults and can be considered good. Similarly, the resulting completion rates for the 3L and 5L were also found to be very high with more than 94% of respondents reporting complete EQ-5D health state information [20, 42–44]. Even though 5L and 3L were consistently close, the 5L did not result in better feasibility properties than the 3L as suggested elsewhere [44, 45]. Nevertheless, these results should be considered in the light of the high proportion of respondents who required help completing the survey, which was around 59%. Of these, almost 70% had both someone reading the questions to them and helped filling in the response, which technically corresponds to an interviewer-based approach. This figure is consistent with other studies using the 3L in older adults, but highlights the increased need of assistance for older people when participating in QoL surveys [46, 47]. Interestingly, missing values were also very low for the WHOQOL-OLD. This seems surprising given that longer measures, such as the AQoL-4D or the SF-36, which are frequently used in samples of older adults, tend to produce more missing values in comparison to the EQ-5D [48, 49]. However, a study by Rolstad et al. [50] found that response burden is not necessarily associated with length of the questionnaire, but with content. Hence, this may explain the better feasibility of the WHOQOL-OLD, which is specifically designed for the use in older respondents. Another important aspect is the mixed evidence with regard to the completion of the EQ VAS. While only 4% of all responses to the EQ VAS were completely missing, we observed a considerable share of respondents (33%) with inadequate responses. Response or comprehension issues with the EQ VAS in older populations were reported before [20, 46, 47, 49]. However, while we were able to extract an EQ VAS rating for more than 90% of the respondents, these ratings seem to be more prone to error given that almost one third failed to respond in accordance with the instructions.

Overall, our test-retest findings suggest good reproducibility of both the 3L and 5L for the index as well as the individual dimensions, with no clear pattern of superiority for either version. According to a recent review from Gottschalk et al. [17] this is the first study assessing test-retest reliability of the 5L in older adults, thus, our reliability findings on the 5L cannot directly be compared with an age-adequate sample. With regard to reliability of the 3L index, our results suggest slightly better reproducibility in comparison to what was found in earlier studies with older adults; however, these included respondents with Alzheimer’s disease and dementia [51–53]. In a broader comparison, our resulting retest statistics agree with those reported in Buchholz et al. [42], but appear at the upper end of the range of reported test statistics. This is likely due to the fact that we aimed to control for a stable health state and retest respondents had a very consistent interval of 14 days between assessments [54]. In comparison to the EQ-5D, evidence with regard to the reproducibility of the WHOQOL-OLD was mixed. While the ICC for the total score was comparable in size to the 3L and 5L index scores, the ICC of the individual facets deviated widely. A similar pattern was observed for the weighted kappa statistics on the 24 WHOQOL-OLD items. The findings from an earlier study deviate from ours, in the sense that calculated ICCs were higher for the individual facets. At the same time, the lower reproducibility for the autonomy and past, present and future activities facets was not shown in the Chinese study [55].

### Strength and limitations

A strength of our study is the high proportion of older respondents even beyond the age of 80 and the good representation of respondents with greater need for care as suggested by the more severe care levels. Our study also adds to the scarce literature on test-retest reliability with a comparatively large retest sample of older care-dependents. However, the convenience sample is a limitation in our study for two reasons. First, the sample is unlikely to be representative, which does not allow to generalise the results towards the entire population. Secondly, the two-staged sampling strategy may have resulted in a sampling bias. Feedback from cooperating institutions suggests that participation was primarily refused due to a lack of interest or a self-perceived health status that was too poor to participate. Hence, this sample may have a positive selection with regard to health status and impairments. Furthermore, it has been discussed that the EQ-5D may be problematic in dementia, which is often prevalent in older adults. Unfortunately, we were not able to control our results for the cognitive status of respondents. But, given the promising results found here and in contrast to the problematic application of EQ-5D in patients with dementia described in the literature, we have to assume that mental conditions were underrepresented in our sample.

## Conclusion

Both the 3L and 5L showed good test-retest and feasibility properties in this sample with high completion rates, few missing values and good reproducibility of the index and individual dimensions. Generally, the EQ-5D descriptive system seems to be sensitive towards greater need for care as classified by the German needs assessment for care, i.e. the resulting care levels. The analysis of relationship between measures indicated that EQ-5D and WHOQOL-OLD assess partially overlapping, but distinct constructs. Hence, we conclude that using the WHOQOL-OLD alongside EQ-5D in this sample added further information on different aspects of QoL from care-dependents. However, researchers should be aware of the high proportion of people needing assistance to complete these measures, which may have important implications for the data collection process in similar samples. Even though neither version of the EQ-5D indicated superiority over the other, proper investigation of measurement aspects of the 5L are rare. Overall, further research is warranted to generalise these findings with additional validation studies in the context of aged care services–with an emphasis on evidence on the 5L to provide a better basis to decide which version of the EQ-5D to pick in clinical or economic evaluation studies in the context of health and aged care.

## Supporting information

S1 Data (XLSX)

## Abbreviations

ANOVA: Analysis of variance
3L: EQ-5D-3L; 5L –EQ-5D-5L; EQ VAS—EQ-5D Visual Analogue Scale
ICC: Intraclass correlation coefficient
QoL: Quality of life
